# Cognitive development and children's perceptions of fruit and vegetables; a qualitative study

**DOI:** 10.1186/1479-5868-4-30

**Published:** 2007-07-09

**Authors:** Gertrude G Zeinstra, Maria A Koelen, Frans J Kok, Cees de Graaf

**Affiliations:** 1 Department of Human Nutrition, Wageningen University, 6700 EV Wageningen, The Netherlands; 2 Department of Communication Science, Wageningen University, 6700 EW Wageningen, The Netherlands

## Abstract

**Background:**

Most children do not meet the recommended guidelines for fruit and vegetable intake. Since preference is an important predictor of intake, more knowledge is needed about children's preferences and about how these preferences develop. As most research about preferences has ignored cognitive development, this study was designed to explore the relation between children's perceptions and preferences for fruit and vegetables and their cognitive development.

**Methods:**

The study population consisted of eight 4–5-year-old children, eight 7–8-year-old children and twelve 11–12-year-old children, recruited via a primary school in Wageningen, The Netherlands. Qualitative in-depth information was obtained by duo-interviews and focus group discussions. A structured guide with questions and game tasks was applied to address different domains in a consistent way.

**Results:**

The developmental progress at the abstraction level was seen in children's reasoning across all domains. Children's preferences expanded and increased in complexity as they moved to a higher age bracket. The most important determinants for liking and disliking shifted from appearance and texture attributes in 4–5-year-olds towards taste attributes in 11–12-year-olds. Children's knowledge of basic tastes increased. Their understanding of health improved as they grew older. The emergence of social norms and perspectives of others as the children grew older was also seen in relation to fruit and vegetables. Child-reported parental strategies to stimulate healthy eating appeared to vary with age in line with cognitive development.

**Conclusion:**

Cognitive development is paralleled by changes in the importance given to the attributes that determine whether a child likes or dislikes fruits and vegetables; children's understanding of and reasoning about health; and parental use of strategies. These developmental differences should be incorporated in programs designed to increase long-term fruit and vegetable intake in children.

## Background

The beneficial effects of eating fruit and vegetables are widely acknowledged [1,2]. However, many children do not meet the recommended guidelines for fruit and vegetable intake [3-5]. The Dutch recommendations for 4–12-year-old children of 150 grams of vegetables and two pieces (≈200 gram) of fruit, are in line with international guidelines [6]. Because food preferences and eating habits established in childhood often persist into adulthood, children are an appropriate group to target in order to positively influence dietary habits [7-9].

In recent years, several studies and programs have been set up to increase fruit and vegetable intake in children [10,11]. Positive changes have been found in knowledge, self-efficacy, skills, awareness, liking and intake. However, real, long-term successes have been difficult to establish [10,11]. To our knowledge, there is one recent study that did show long-term effects of a one-year free school fruit programme on children's fruit and vegetable consumption [12].

Preference is an important predictor of children's food intake [8,13-15]. For vegetables in particular, children's preference is low [15-18]. Therefore, to stimulate fruit and vegetable consumption among children, more should be known about their preferences, how these develop and how they can be influenced.

Most research about children's preferences does not take the possible role of children's cognitive development into account. Cognitive development represents "the sequence of changes that occur to the cognition of a person as they mature" [19]. Cognition refers to "the mental processes responsible for perception, attention, learning, memory, thought and communication" [20]. The aim of this study is to explore the relation between children's cognitive development and their perceptions of, and preferences for, fruit and vegetables.

### Cognitive development and nutrition behaviour

Jean Piaget developed a cognitive development model with four successive stages: sensory motor period (0–2 years), pre-operational stage (2–7 years), concrete operational stage (7–11 years) and the formal operational stage (11–15 years). Along these stages, children's thinking changes from concrete to abstract, they develop the ability to replace overt actions by mental representations, egocentrism and centration diminishes, children develop more eye for detail, their information processing capacities increase, and their problem solving becomes more and more advanced [21-24].

For our study, we selected three age categories to study a broad range of cognitive development: 4–5-year-old, 7–8-year-old and 11–12-year-old children. Table 1 summarizes the cognitive developmental differences between the three age groups.

**Table 1:** Overview of general and nutrition related cognitive characteristics of children

**Pre-operational stage**	**Concrete operational stage**	**Formal operational stage**
Limited information processors	Cued processors	Strategic processors
Egocentric	Aware of perspective of another	Able to consider different perspectives
Focused on one attractive external characteristic	Focused on two or more functional and underlying attributes	Focused on multiple functional and underlying attributes
Decisions based on salient perceptual attributes	Decisions more flexible and thoughtful	Decisions more strategic
Do not consider transformations	See intermediary processes	Eye for detail
Concrete thinking	Thinking more logical, but concrete	Abstract thinking
Pre-logical thinking	First type of causality thinking	Logical reasoning
No distinction between foods and snacks	Distinction between foods and snacks	*
Ingested foods not changed in stomach	Ingested foods are changed somehow in the stomach	*
Can mention healthy foods, but not explain why it is healthy	Healthy foods make you strong, healthy and grow (do not know how)	*
Brand preferences based on perceptual and affective attributes	Brand preferences based on cognitive attributes	*
Rejection based on distaste, danger or ideational	Rejection based on distaste, danger, disgust or inappropriateness	Rejection based on distaste, danger, disgust or inappropriateness
No idea of contamination	Basic idea of contamination	Full adult idea of contamination

* No data available for this age group; these studies did focus on children in the pre-operational and concrete operational stage

A few studies in the area of nutrition behaviour have taken cognitive development into account. Contento's [25] investigation about how children think about food and eating revealed that children in the pre-operational stage did not make a distinction between foods and snacks, whereas children in the concrete operational stage did. Pre-operational children believed that the ingested food went into the stomach and did not change in the body. Concrete operational children understood that food was changed somehow in the stomach. Pre-operational children could mention foods that were healthy, but they could not explain why. Concrete operational children could tell that food made you strong, healthy and made you grow, but they could not explain why or how this occurred.

Bahn [26] studied brand preferences and brand discriminations. Affectively based attributes, such as liking the taste or liking the colour of the package, were dominant in pre-operational and concrete operational children when they were distinguishing brands. Regarding preferences, concrete operational children focussed more on cognitively based attributes, such as healthiness and adultness, than pre-operational children.

It is interesting to note Rozin et al.'s perspective in this context. They showed that there is a gradual emergence of different categories of food rejections as the child matures [27,28]. Very young children of 1–21/2 years old accept almost all kind of edible and inedible substances. The first rejection category to appear is distaste; disliked products are rejected. Secondly, rejections based on danger appear. This means that products are rejected because negative consequences of ingestion are expected. The third rejection category is based on the idea of what something is or where it comes from (ideational). This category can be split into disgust, and inappropriateness. Disgust means that the association with the food product is averse, whereas inappropriateness means that the food product is not considered to be a food. It is not until the age of 7 that children differentiate between disgust and inappropriateness. The idea of contamination appears gradually between the ages of 31/2 years and 12 years. A food is contaminated when even a trace amount of a disgusting or inappropriate product has been or is present in the food [27,28]. This development of rejection is in line with the development of the child. Between the ages of 2 and 7, children become more independent eaters and they have to learn which foods are edible and which foods are not [21,28].

The nutrition studies above show that children in distinct cognitive stages think, decide and perceive food topics differently. The ideas children have about specific foods can influence their preferences, their willingness to taste and their whole eating experience [29]. Consequently, these different thoughts, perceptions and decision strategies may significantly impact on interventions aimed at changing food preferences and intake. Because most current approaches have not been effective in establishing long-term changes in fruit and vegetable consumption, cognitive development may be a promising field for achieving such changes; new approaches that are appropriate with regard to cognitive development will correspond closely with the children's natural development. In this study, we explored how the differences in cognitive development relate to children's perceptions of, and preferences for, fruit and vegetables. On the basis of the cognitive development theories, we expect that the number of cognitions about fruit and vegetables will increase as children grow up and that these cognitions will increase in complexity and abstraction.

## Methods

### Participants

Participants were recruited via a primary school in Wageningen, The Netherlands. Three age groups were included in the study, each age group representing a different stage of cognitive development: 4–5-year-old children (pre-operational stage: group A), 7–8-year-old children (concrete operational stage: group B) and 11–12-year-old children (formal operational stage: group C). The age groups correspond to the first, fourth and last grade of primary school in The Netherlands.

In group A, the two youngest and two oldest boys and the two youngest and two oldest girls who had permission to participate joined the study. In group B, four boys and four girls were selected by lottery. In group C, three groups were formed: one group of four girls, one group of four boys and a mixed group of two boys and two girls. The principal of the school and the parents of the children provided their informed consent.

### Qualitative research

Because little is known about the relation between cognitive development and children's preferences, a qualitative approach was used to gain more insight and understanding about this relationship [30,31]. Qualitative research provides the opportunity to explore new topics and directions; thereby using the respondents' own words to give meaning to their world [30].

In our study we used focus group interviews for group C children. The purpose of a focus group is to elicit respondents' personal perceptions of a defined area of interest through carefully planned, semi-structured discussions [32]. Respondents can react and build upon other members' responses, and this in turn leads to more thoughtful and in-depth information [31,33,34]. In addition, it is possible to ask follow-up questions for clarification [31]. Focus groups are especially valuable for obtaining data from children [31-33].

Duo-interviews were held with the younger children in groups A and B. Children of this age need more assistance and have fewer communication skills. That is why duo-interviews are often used for this age group in market research [35]. Two children of the same sex and same age are interviewed together. Advantages are that the situation for the children is more natural and less scary than if they were alone, they speak more freely when they are with a child of the same age and same sex, and the attention of the interviewer is not all the time on one child. When the children know each other, there is another advantage: the children can point each other to untrue statements. The children can build upon each other's responses, but do not have to process responses of many other group members. Although the children can interfere with each other, we expected that the advantages of this method would compensate for the disadvantage of interference. Besides, care was taken to prevent dominant children from biasing the results: the moderator used different questioning strategies to include all participants' opinions and, if there was a dominant child in the group, the moderator took care to invite the less dominant child to give his or her opinion before the dominant child.

### Procedure

In May and June 2005, three focus group sessions (four children in each) were held with group C. Each session took approximately 90 minutes. In group B, four duo-interviews were held, each lasting approximately 75 minutes. All four duo-interviews in group A took about 60 minutes. Because young children have a short attention span, the 4-year-old children were interviewed twice, for half an hour each time, on two different days. The interviews were held in a separate, quiet room at school during lessons with no parents or teacher present. All interviews were recorded and video taped. A research assistant was present to take notes, keep track of the time, record non-verbal information and control the video and sound recording. On finishing the conversation, each participant received a small present.

### Question route

As advised by Morrison-Beedy et al. [34] for multiple group comparison, a structured interview guide was devised to ensure consistency in data collection. This guide was applied in the focus group discussions as well as in the duo-interviews and ensured a proper introduction, which is very important to make the children feel at ease. The rules of conversation were explained to them, as well as confidentiality, anonymity, recording of the sessions, and the fact that there are no wrong answers [21,34].

In addition to questions, game tasks and fruit tasting were included, to get richer information and to keep the children concentrating. Health was the final topic introduced during the conversations to prevent the children from focussing on health during the whole interview. An expert in child interviewing checked the interview guide and made suggestions for improvement. The guide was pilot tested on three children aged 5–6 years. Improvements were made to ensure that the questions and game tasks were clear and understandable.

To get a better understanding of children's preferences and their perceptions, thoughts, learning and communication about fruit and vegetables, diverse topics were addressed during the conversations: spontaneously probed preferences and dislikes, attributes leading to liking and disliking, categorization of fruit and vegetables, tasting fruits, healthy eating strategies, appropriate eating situations for fruit and vegetables, free associations and the concept of health. To make it easier and concrete for the children, various real fruits and vegetables were brought to the sessions. Picture cards were used to assist the children in pointing out appropriate eating times and occasions (six eating times: breakfast, morning break, lunch, afternoon, dinner and evening + six occasions: home, school, party, sport, being with friends and TV/computer). Seven fruits were chosen for tasting. We included fruits that varied widely in their taste, appearance, frequency of use and familiarity: strawberry, apple, mango, papaya, kiwi, grapefruit and lemon. Six vegetables were chosen based on the same arguments, but these were not tasted: carrot, cauliflower, egg plant, red peppers, French beans and chicory. The question route can be found in Table 2.

**Table 2:** Question route

Spontaneously probed (dis)likes	"If you think about food, what do you like best in the world?" "If you think about food, what do you think is the worst food in the world?"
Attributes responsible for (dis)liking	"What is it that makes this product so nice/awful?"
Categorization of fruit & vegetables	The children were invited to group the 15 fruit and vegetable products according to their opinion. They could choose how many groups they wanted to make.
Tasting seven pieces of fruit	The children were asked to taste the fruit pieces (in random order) and to tell what they liked or disliked about it. The characteristics of the product were also discussed.
Healthy eating strategies	"If you are served a food that you do not like, what happens then?" "What do your parents say about fruits/vegetables?"
Appropriate eating situations	"Which picture depicts the most appropriate moment for eating fruit/vegetables?" "Which picture depicts the most appropriate occasion to eat fruit/vegetables?"
Free associations	Associations and images for fruit and vegetables were explored by questions, free associations and game tasks about coolness, boringness, and appropriate target population for fruit and vegetables.
Concept of health	"Can you explain what health means?" Then the children were shown five pictures of products: grapes, leek, French fries, tart and candies. For each product they were asked: "Do you think this product is healthful?" "Why do you think that it is healthful/not healthful?"

### Data analysis

The recorded interviews were transcribed by the interviewer and the assistants. The interviewer checked the transcript with the video records, in order to add non-verbal information. We developed a coding framework based on the research aims, the interview guide and findings in the literature. The qualitative data analysis package N6 from QRS International (version 2002) was used to code and organise the data systematically. Significant statements were coded with a label and corresponding statements were coded with the same label. Based on the children's statements, we chose an appropriate term for each label to summarize the statements within a category. This organisation of data into different categories and sub-categories assisted a more effective comparison of the groups.

Initially, data analysis was carried out by the first author, who was physically present in the room when the duo-interviews and focus groups were conducted, as is advised by Krueger and Casey [32]. First, the data within an age category were analysed. Subsequently, the data of the three age groups were compared thoroughly to detect patterns and find similarities and differences. In discussion sessions with all authors of the manuscript, the results of the analyses were repeatedly and thoroughly discussed. In addition, the results were presented and discussed with other researchers and with external experts in the field of research with children and taste, to check interpretations and conclusions. Key concepts and patterns are discussed below.

## Results

### Preferences, dislikes and attributes leading to liking and disliking

Table 3 summarizes children's spontaneously mentioned preferences and dislikes. The results show that children's preferences and dislikes expand as they grow up. Group A spontaneously mentioned soft, high-energy foods, such as pancakes and French fries, and sweet fruits as their most preferred food. Group B mentioned composite dishes and meat, besides soft, high-energy foods and fruits. Composite dishes are food dishes with various ingredients, such as pizza or vegetable pie. Spontaneously mentioned favourite foods of many children in group C were composite dishes with vegetables as the principle component. The other preferred foods were comparable to groups A and B.

**Table 3:** Preferred and disliked food groups together with the top 3 of most often mentioned attribute categories based on the children's reasons for liking and disliking

	4–5 years	7–8 years	11–12 years
Preferences	Soft, high-energy foods Fruit	Composed dishes Soft, high-energy foods Fruit	Vegetable dishes Composed dishes Soft, high-energy foods Fruit
Dislikes	Bitter vegetables	Bitter vegetables Bland vegetables	Bitter vegetables Sour vegetables Bland vegetables
Basis for liking	1. Texture 2. Taste 3. Preference	1. Texture 2. Taste 3. Sweetness 3. Sourness	1. Taste 2. Texture 3. Preference for topping
Basis for disliking	1. Texture 2. Taste 3. Appearance	1. Taste 2. Sourness 3. Texture	1. Sourness 2. Bitterness 3. Negative (expected) experiences

When asked about the food they disliked most, almost all children spontaneously mentioned a vegetable. Group A referred to bitter vegetables, such as Brussels sprouts, spinach and chicory. In group B, children indicated also vegetables with a more bland taste, French beans for example. Children in group C mentioned bitter, sour and bland tasting vegetables as their least favourite foods.

In Table 3, also the reasons for liking and disliking provided by the three age groups are summarized. The most important attributes for liking in group A were based on texture, taste and preference ("I just like it"). Additional reasons for liking in group B were based on more specific tastes: sweetness and sourness. Preference based reasons were mentioned less often than in group A. *Familiarity of the taste *and *liking everything about the product *were new reasons compared to group A. The most important reason for liking in group C was a good taste, followed by texture and preference for topping (For example: the sauce on cauliflower). Some children said they liked saltiness or bitterness. These attributes did not come out in groups A and B.

Important reasons for disliking in group A were based on texture, taste and appearance. Reasons for disliking in group B were derived from taste in general, followed by sourness and texture. Disliking in group C was founded on specific tastes such as sourness and bitterness and negative (expected) experiences (For example: "It makes me feel sick" or "It feels like spittle").

### Perception of fruit and vegetables

When the children were asked to make groups of different fruit and vegetable products, the youngest children made groups based on concrete characteristics: colour and shape. For example the lemon and grapefruit were put together, because they are both yellow. A few children in group B based their categorization on concrete characteristics as well. The others used abstract characteristics: a liking dimension or the dimension fruit versus vegetables. All children in group C used abstract characteristics to categorize the products: "liking" or "fruit versus vegetables" or "a mixed dimension", which included frequency of use combined with liking or with fruit versus vegetables.

When the children were asked to explain whether fruits or vegetables were appropriate for adults and/or for children, different perspectives were found. Groups B and C considered fruit to be appropriate for adults as well as for children. Children in group A, however, made a distinction between tasty and non-tasty products. They mentioned that tasty fruits or vegetables were for both children and adults. Non-tasty products were considered appropriate only for adults. Groups A and B mentioned their own preference and physical growth ("helps you grow") as arguments for making the distinction between adult and child food. Healthfulness ("It is healthy") and social norm arguments ("Everybody eats it") emerged in groups B and C.

### Knowledge of tastes

The older the children were, the more comprehensive was their understanding of the basic tastes. Children in group A used salty in the correct way. They did not know what bitter was. Although the children had some understanding of the tastes sour and sweet, they had difficulties in labelling the products with these terms. Many children in groups A and B labelled a lemon as sweet. In group B, sourness was used in the correct way, and a few children were familiar with the term bitter. It was not until group C that the children used salt, sour, sweet and bitter in the correct way.

### Associations and images

To find out whether the image of fruit and vegetables is a barrier to consumption, children were asked whether fruit and vegetable products were cool or boring. These terms were difficult for group A. Groups B and C stated that these terms were not really appropriate terms: fruit and vegetables are neither cool nor boring.

It was surprising to see that the younger the child, the more enthusiastic and happy the child was when it saw a highly liked product, such as strawberries for most children. Free associations for fruit and vegetables were quite difficult for the children. With increasing age, children made more abstract and functional associations.

### Appropriate times and occasions

Children's ideas about appropriate eating situations for fruit and vegetables are shown in Table 4. All groups considered lunch and the afternoon as appropriate times for eating fruit. Strawberries were the only type of fruit thought to go well with breakfast. Group B mentioned that fruit as a dessert after dinner was possible. Only group C considered the evening as a possible time for eating fruit. When asked about an appropriate time for vegetables, children of all ages agreed that dinner was the right time.

**Table 4:** Summary of children's statements about appropriate fruit and vegetable-eating situations and parental and child-invented healthy eating strategies

	4–5 years	7–8 years	11–12 years
Appropriate times for eating fruit	Fruit for lunch or afternoon Vegetables for dinner	Fruit in afternoon, for lunch or dinner dessert Vegetables for dinner	Fruit in afternoon, evening, lunch or when you feel like eating it Vegetables for dinner
Appropriate occasions for eating fruit	Home School Party	Home Being with friends* Sport *	Home Being with friends* Sport * TV/computer *
Arguments for appropriate times and occasions based on	Features of picture Own behaviour	Own behaviour Parental & school rules Busyness status Features of picture *	Own behaviour Social norm Availability Busyness status Function of fruit
Parental healthy eating strategies	Moderation Health arguments Instrumental rewarding Taste masking	Moderation Health arguments Taste Masking	Moderation Health arguments Parental cooking effort
Child-invented strategies to cope with vegetables	Not mentioned	Various strategies present	Various strategies present

* Stated by 50% of the children in this age group

All age groups associated the home environment with eating fruit. Only group A associated eating fruit with school and a party. In group B, half of the children agreed that sport and being with friends were appropriate occasions for fruit, besides home. This was similar to group C, but at this age half of the children also saw the computer/TV as a good occasion for eating fruit. Group C stated that fruit and vegetables were too healthy for a party. A party was associated with eating candy and other "unhealthy stuff".

The three age groups used different arguments when deciding on appropriate times or occasions. Group A relied on their own behaviour or on the features of the picture cards. Group B referred to their own behaviour, parental and school rules or the opportunity for eating. A few children used the features of the picture cards in their argumentation for appropriate times. In group C, social norms emerged in reasoning about appropriate times. The children took into account what they had seen their peers doing or not and they were aware of a general norm ("It is not common to do this"). In addition, they used arguments relating to their own behaviour, the availability of food, the (time) opportunity for eating and the functions of fruit (energy for example).

In summary, children of all ages had fixed ideas about appropriate times and occasions for eating fruit and vegetables. With increasing age, the children saw more opportunities for eating fruit. In addition, older children used a broader range of arguments and their arguments were more abstract.

### Parent and child healthy eating strategies

The conversations made clear that almost all parents try to influence the eating behaviour of their child. The children were very well aware of the rules and strategies their parents apply in relation to eating. To promote healthy eating, parents in all age groups used "moderation" and "health" arguments. Moderation refers to children's statements where they indicated that the intake of some foods was restricted to certain times of the day or week. However, there were also differences found between the three age groups (See Table 4). In group A, instrumental eating was used more often than in the other age groups. Instrumental eating means that the children are promised a reward if they eat well [36]. Another often-used strategy was permission to use apple sauce in combination with disliked vegetables, which is a form of taste masking. This tactic was applied quite often in group B too. Children in this age group said that they invented their own ways of dealing with disliked products: they made vegetables very flat and added a lot of apple sauce or they ate ten very small bites, so they actually ate five normal bites. In group C, adding apple sauce was a less used strategy. In this age category, parents mentioned the effort they had put into cooking the vegetables as an argument to get the children to eat them. All children in group C had invented their own creative strategy to cope with eating disliked vegetables, such as squeezing their nose, finishing first the non-tasty food or adding ketchup to the vegetables.

### Concept of health

A clear trend was seen for the concept of health. Young children could not describe health. The older the children, the more comprehensive and abstract the concept. Most of the 4–5-year-olds could not categorize products correctly into healthy or not healthy. They used concrete and simple "rules" to categorize products as healthy or not. The most popular justification rule was a food-colour link, such as "It's healthy, because it's green". This fits for the leek, but not for a green candy. Second and third most popular arguments were food-health links (food related to healthy food group such as "It is fruit and fruit is healthy") and preference links ("It's healthy, because I like it"). The other two age groups could correctly categorize products into healthy or not. In groups B and C, food-health and food-nutrient links (food classification linked to its provision of a specific nutrient such as "It is healthy, because it contains vitamin C") were most popular justification categories. Social influence ("family says" or "others say") and general knowledge ("I just know") were the third and fourth most often mentioned.

## Discussion

This study indicates that the stage of children's cognitive development plays a role in their preferences for, and perceptions of, fruit and vegetables. As children mature, their cognitions relating to fruit and vegetables increase in number and become more abstract.

Although cognitive development as a viewpoint from which to study fruit and vegetable *preferences *is new, our findings are not incompatible with previous research. Age related differences in preferences have been found in other studies [8,37]. An interesting finding from our study was that cognitive development is related to the attributes children consider when evaluating products. Young children focus on appearance and texture, whereas older children focus on taste aspects. Rose et al. [38] found similar results with sensory preferences for meat. For 6–7-year-old children, mouth feel characteristics were most important for liking, whereas in 10–11-year-olds taste and smell were most important. The diminished importance of textural attributes is possibly due to children's development of their teeth and jaws [29,39]. Szczesniak [39] stated that texture would be especially important for disliking products, but in our study it was also the most important attribute for liking among the two youngest age groups.

Young children could tell whether they liked or disliked the taste of a product, but could not identify the specific taste. This finding is in line with the study of Liem et al [40] where 4-year-old children could indicate which solution they preferred but failed to distinguish sweetness intensities during discrimination tests. In our study, the 4–5-year-old children knew and could properly use the taste salt, but not the other basic tastes. Older children had an improved understanding of the four basic tastes and, consequently, were more specific about taste when talking about likes and dislikes.

A shift from appearance to more functional attributes was found in children's reasoning with regard to preferences, the appropriate situations to eat fruit and vegetables, and healthiness. In other studies it has been found that pre-operational children focus on the most striking attributes that catch the eye, whereas older children use more functional and underlying attributes [23,41].

One aspect of cognitive development is an increase in the level of abstraction [21,22]. This development was clearly seen in children's improved understanding of health and the basic tastes, the shift in categorization from concrete categories to abstract categories, the expansion of abstract associations, and more abstract arguments concerning healthiness. Furthermore, the arguments for appropriate occasions were very concrete in groups A and B, whereas this was not the case in group C. The reduction of egocentrism [21,22] was reflected in the emergence of social norms and consideration of others' behaviour in the older age group in relation to their argumentation for appropriate eating times.

Roos [42] found that 9–11-year-old children could correctly identify which foods are considered healthy, a finding that is in line with ours. As in the findings of Hart et al. [43] among 7–11-year-old children, food-nutrient and food-health links were most often used in groups B and C as an explanation for the healthiness of a food. In our study, only pre-operational children mentioned preference links as an explanation for healthiness. This is in contrast to the findings of Hart et al. [43], where preference links were especially used by the older boys (10–11 years).

Parents play an important role in the nutrition behaviour of children [44-46]. Hart et al. [43] showed that parents used different rules depending on the age of the child. Food deals were more frequently reported by younger children (7–8 years) compared to older children (10–11 years). Although the age range is somewhat different, these findings are in agreement with our findings, where instrumental rewarding, which is comparable to a food deal, was used in respect of the youngest children but disappeared as children grow up. Probably, parents use instrumental rewarding, because it is a concrete strategy for the child ("If you eat your vegetables, you will get a candy"). The finding that parental effort is used as an argument in the oldest age group appears to fit their cognitive capacities. At this age, children are less egocentric [21,22], they can see another's perspective [22,23], and have a better understanding of value [23]. So children of this age can understand this argument. The strategies that children in group B invent to cope with eating vegetables reveal their emergent idea of conservation [22].

### Limitations

Although a qualitative approach is the best method to explore a new topic, the limitations should be acknowledged. A small number of children participated in the conversations. Therefore it is not easy to generalize to a larger and broader population [31]. Further research is needed to confirm our findings.

Qualitative research is sometimes criticized for being low in both reliability and validity because of the subjective interpretation of results [31,32]. Several different actions were taken in this study to ensure reliability and validity. We used accepted systematic procedures for data collection, data handling and data analysis. The fact that children were assured that there were no wrong answers and that we did not finish their responses for them supports validity [47]. As advised by Morrison-Beedy et al. [34], the conversations were discussed immediately afterwards by the moderator and research assistant. Major topics, confusing and conflicting data were discussed. The analyses and interpretations were thoroughly discussed with the co-authors, with other researchers and with experts in research with children and taste. In addition, the comparison of our results with other findings in the literature strengthens evidence [47]. We have been very careful with interpretation and are confident that the findings are an accurate reflection of what the children said.

Another limitation is that we did not measure cognitive development. It is true that children develop at different rates, and this can result in differences within an age group. However, on the basis of cognitive development theories we are convinced that the differences in cognitive development between children of distinct age groups are larger than the differences between children within an age group. Besides, it would have been very impractical to measure the whole concept of cognitive development, as this is enormously time consuming and would have been a heavy burden for the children.

### Practical implications and future research

A great advantage of our study method is that we found important practical implications and fruitful directions for future research that would have been missed with a quantitative approach. In our study, vegetables came out as least favourite food in all age groups. This barrier needs to be tackled in order to increase children's vegetable consumption. A promising finding was that fruits (especially apples and strawberries) were liked and that almost all children liked at least one vegetable. It was also positive that children considered fruit and vegetables as food for themselves as well as for adults; it would be a barrier if they perceived it only as adult food. It is often thought that fruits and vegetables are not cool enough for children. However, our study found that this did not play a role in children's consumption.

A reason often mentioned for eating fruits and vegetables at a particular time was "Because I eat it at that time". So if we teach children to have more fruit and vegetable eating times during the day, we could increase their intake. In our study, just a few children in group C associated fruit with the computer or TV. Because children spend many hours in front of the TV or computer nowadays [48], making this activity a fruit or vegetable eating time would be a first step towards improving children's diet.

In group C, it became apparent that the children did not think of cucumber and tomato as vegetables. When the research assistant mentioned that cucumber and tomato are also considered vegetables, then the children suddenly saw more time opportunities for eating vegetables: slices of tomato on bread during lunch or a piece of cucumber during the morning break at school. This finding could be very valuable in promoting vegetable intake by increasing the number of daily vegetable eating times.

In our study, older children were more specific about the preferred preparation of vegetables, and young children valued textural attributes and appearance more, whereas older children valued taste aspects more. Thus by using different preparation methods to match the right attributes to the desires of each specific age group, we might be able to change children's fruit and vegetable preferences and consequently their intake.

A very surprising finding in our study was that the youngest children argued that foods are healthy, because they taste nice. Research has shown that children associate healthy with distaste [49-51]. However, the age of the children in these studies was nine years and older, whereas the children in this group in our study were 4–5 years. It may be that young children associate healthy with tasty through the connecting term "good"; tasty food is good and being healthy is also good. However, at a certain age point, there seems to emerge a differentiation: not all healthy foods taste good. It would be interesting to investigate at what age this negative change in association occurs and how this change comes about.

In concordance with Hart et al. [43], our results indicated that parents do not take many positive actions. The children should eat healthily, are restricted to specific foods and are often persuaded to eat fruit and vegetables because they are healthy. It is not clear whether parents did not apply more positive strategies or whether more positive strategies were just not reported by the children. If parents use many negative strategies for healthy products, then this may be a reason why children develop a negative taste association for these healthy foods [36]. Our study suggests that children's cognitive development influences the strategies that parents use to shape the eating behaviour of children. It would be very interesting to investigate this interaction between parents and children further, together with the effects of this interaction.

## Conclusion

This study is the first step in understanding how cognitive development and preferences are interrelated. Differences in cognitive development are reflected in changes in attribute importance in relation to liking and disliking fruits and vegetables, in children's understanding of, and reasoning about, health, and in the child-reported parental use of strategies. Further research should focus on the role of parental strategies in their children's preferences and intake of fruit and vegetables, children's underlying reasons for liking and disliking in different age groups, and how the concept of health develops during childhood years. For optimal results in the long term, children's thoughts, perceptions, decision arguments and abstraction capacities should be taken into account in the development of interventions for promoting fruit and vegetable intake among children.

## Competing interests

The author(s) declare that they have no competing interests.

## Authors' contributions

GGZ, MAK and CG contributed to the design of the study. GGZ coordinated and carried out the study in practice. GGZ was in charge of the transcription process and did the data analyses, with critical input from MAK, CG and FK. GGZ wrote the original draft and incorporated the comments on the manuscript from all other authors. All authors have read and approved the final manuscript.

## References

[B1] Hung HC, Joshipura KJ, Jiang R, Hu FB, Hunter D, Smith-Warner SA, Colditz GA, Rosner B, Spiegelman D, Willett WC (2004). Fruit and vegetable intake and risk of major chronic disease. J Natl Cancer Inst.

[B2] WHO (2002). The World Health Report.. Reducing risks, promoting healthy life.

[B3] Gibson EL, Wardle J, Watts CJ (1998). Fruit and vegetable consumption, nutritional knowledge and beliefs in mothers and children. Appetite.

[B4] Voedingscentrum (1998). Zo eet Nederland 1998.

[B5] Yngve A, Wolf A, Poortvliet E, Elmadfa I, Brug J, Ehrenblad B, Franchini B, Haraldsdottir J, Krolner R, Maes L, Perez-Rodrigo C, Sjostrom M, Thorsdottir I, Klepp KI (2005). Fruit and vegetable intake in a sample of 11-year-old children in 9 European countries: The Pro Children Cross-sectional Survey. Ann Nutr Metab.

[B6] WHO (2003). Diet, Nutrition and the prevention of chronic diseases. Report of a Joint WHO/ FAO Expert consultation.. WHO Technical Report Series 916.

[B7] Nicklaus S, Boggio V, Chabanet C, Issanchou S (2004). A prospective study of food preferences in childhood. Food Qual Pref.

[B8] Nu CT, MacLeod P, Barthelemy J (1996). Effects of age and gender on adolescents' food habits and preferences. Food Qual Pref.

[B9] Skinner JD, Carruth BR, Wendy B, Ziegler PJ (2002). Children's food preferences: a longitudinal analysis. J Am Diet Assoc.

[B10] Blanchette L, Brug J (2005). Determinants of fruit and vegetable consumption among 6-12-year-old children and effective interventions to increase consumption. J Hum Nutr Diet.

[B11] Knai C, Pomerleau J, Lock K, McKee M (2006). Getting children to eat more fruit and vegetables: A systematic review. Prev Med.

[B12] Bere E, Veierod M, Skare O, Klepp KI (2007). Free school fruit - sustained effect three years later. International Journal of Behavioral Nutrition and Physical Activity.

[B13] Bere E, Klepp KI (2005). Changes in accessibility and preferences predict children's future fruit and vegetable intake. Int J Behav Nutr Phys Act.

[B14] Domel-Baxter S, Thompson WO (2002). Fourth-grade children's consumption of fruit and vegetable items available as part of school lunches is closely related to preferences. J Nutr Educ Behav.

[B15] Perez-Rodrigo C, Ribas L, Serra-Majem L, Aranceta J (2003). Food preferences of Spanish children and young people: the enKid study. Eur J Clin Nutr.

[B16] Diehl JM (1999). Food preferences of 10- to 14-year-old boys and girls. Schweiz Med Wochenschr.

[B17] Domel SB, Baranowski T, Davis H, Leonard SB, Riley P, Baranowski J (1993). Measuring fruit and vegetable preferences among 4th- and 5th-grade students. Prev Med.

[B18] Edwards JS, Hartwell HH (2002). Fruit and vegetables--attitudes and knowledge of primary school children. J Hum Nutr Diet.

[B19] www.nelmh.org Definition Cognitive Development. http://www.nelmh.org//page_view.asp?c=15&did=1313&fc=007010005.

[B20] www.nimh.nih.gov Definition Cognition. http://mentalhealth.about.com/library/rs/blcog.htm.

[B21] Delfos MF (2003). Ontwikkeling in vogelvlucht. Ontwikkeling van kinderen en adolescenten..

[B22] Flavell JH, Piaget A, McClelland DC (1963). The developmental psychology of Jean Piaget. The university series in psychology.

[B23] Roedder-John D (1999). Consumer Socialization of Children: A Retrospective Look at Twenty-Five Years of Research. J Consum Res.

[B24] Schaffer HR (2003). Introducing child psychology.

[B25] Contento IR (1981). Children's thinking about food and eating - A Piagetian-based study. J Nutr Educ.

[B26] Bahn KD (1989). Cognitively and perceptually based judgments in children's brand discriminations and preferences. J Bus Psychol (Historical Archive).

[B27] Fallon AE, Rozin P, Pliner P (1984). The child's conception of food: the development of food rejections with special reference to disgust and contamination sensitivity. Child Dev.

[B28] Rozin P, Hammer L, Oster H, Horowitz T, Marmora V (1986). The child's conception of food: differentiation of categories of rejected substances in the 16 months to 5 year age range. Appetite.

[B29] Oram N (1994). Children's eating experiences could differ from those of adults. Appetite.

[B30] Maso I, Smaling A (1998). Kwalitatief onderzoek: praktijk en theorie.

[B31] Stewart DW, Shamdasani PN (1990). Focus groups : theory and practice. Series title Applied social research methods series (vol 20).

[B32] Krueger RA, Casey MA (2000). Focus groups : a practical guide for applied research..

[B33] Horner SD (2000). Using focus group methods with middle school children. Res Nurs Health.

[B34] Morrison-Beedy D, Cote-Arsenault D, Feinstein NF (2001). Maximizing results with focus groups: moderator and analysis issues. Appl Nurs Res.

[B35] IPM-Kidwise (2000).

[B36] Birch LL, Birch D, Marlin DW, Kramer L (1982). Effects of instrumental consumption on children's food preference. Appetite.

[B37] Cooke LJ, Wardle J (2005). Age and gender differences in children's food preferences. Br J Nutr.

[B38] Rose G, Laing DG, Oram N, Hutchinson I (2004). Sensory profiling by children aged 6-7 and 10-11 years. Part 2: a modality approach. Food Qual Pref.

[B39] Szczesniak AS (1972). Consumer awareness of and attitudes to food texture. J Texture Stud.

[B40] Liem DG, Mars M, de Graaf C (2004). Consistency of sensory testing with 4- and 5-year-old children. Food Qual Pref.

[B41] Valkenburg PM, Cantor J (2001). The development of a child into a consumer. J Appl Dev Psychol.

[B42] Roos G (2002). Our bodies are made of pizza -- Food and embodiment among children in Kentucky.. Ecol Food Nutr.

[B43] Hart KH, Bishop JA, Truby H (2002). An investigation into school children's knowledge and awareness of food and nutrition. J Hum Nutr Diet.

[B44] Bourcier E, Bowen DJ, Meischke H, Moinpour C (2003). Evaluation of strategies used by family food preparers to influence healthy eating. Appetite.

[B45] Fisher JO, Birch LL (1999). Restricting access to palatable foods affects children's behavioral response, food selection, and intake. Am J Clin Nutr.

[B46] Wardle J, Carnell S, Cooke L (2005). Parental control over feeding and children's fruit and vegetable intake: how are they related?. J Am Diet Assoc.

[B47] Bender DE, Ewbank D (1994). The focus group as a tool for health research: issues in design and analysis. Health Transit Rev.

[B48] Wright JC, Huston AC, Vandewater EA, Bickham DS, Scantlin RM, Kotler JA, Caplovitz AG, Lee JH, Hofferth S, Finkelstein J (2001). American children's use of electronic media in 1997: A national survey. J Appl Dev Psychol.

[B49] Dixey R, Sahota P, Atwal S, Turner A (2001). Children talking about healthy eating: data from focus groups with 300 9-11-year-olds. Nutrition Bulletin.

[B50] McKinley MC, Lowis C, Robson PJ, Wallace JMW, Morrissey M, Moran A, Livingstone MBE (2005). It's good to talk: children's views on food and nutrition. Eur J Clin Nutr.

[B51] Wardle J, Huon G (2000). An experimental investigation of the influence of health information on children's taste preferences. Health Educ Res.

